# Supplementary material for: Compound mortality impacts from extreme temperatures and the COVID-19 pandemic
Source: Nat Commun. 2024 May 23;15:4289. doi: 10.1038/s41467-024-48207-2 (PMC11116452; doi:10.1038/s41467-024-48207-2)
Supplement: Supplementary file 1 — Supplementary Information [file 41467_2024_48207_MOESM1_ESM.pdf]

Compound mortality impacts from extreme temperatures and the COVID-19 pandemic

Y. T. Eunice Lo[1,2,*], Dann M. Mitchell[1,3] and Antonio Gasparrini[4]

[1] Cabot Institute for the Environment, University of Bristol, UK

[2] Elizabeth Blackwell Institute for Health Research, University of Bristol, UK

[3] School of Geographical Sciences, University of Bristol, UK

[4] Environment & Health Modelling (EHM) Lab, Department of Public Health Environments and Society, London School of Hygiene & Tropical Medicine, London, UK

Supplementary information

- Figure S1
- Table S1
- Table S2
- Figure S2
- Figure S3
- Figure S4
- Figure S5
- Figure S6
- Figure S7

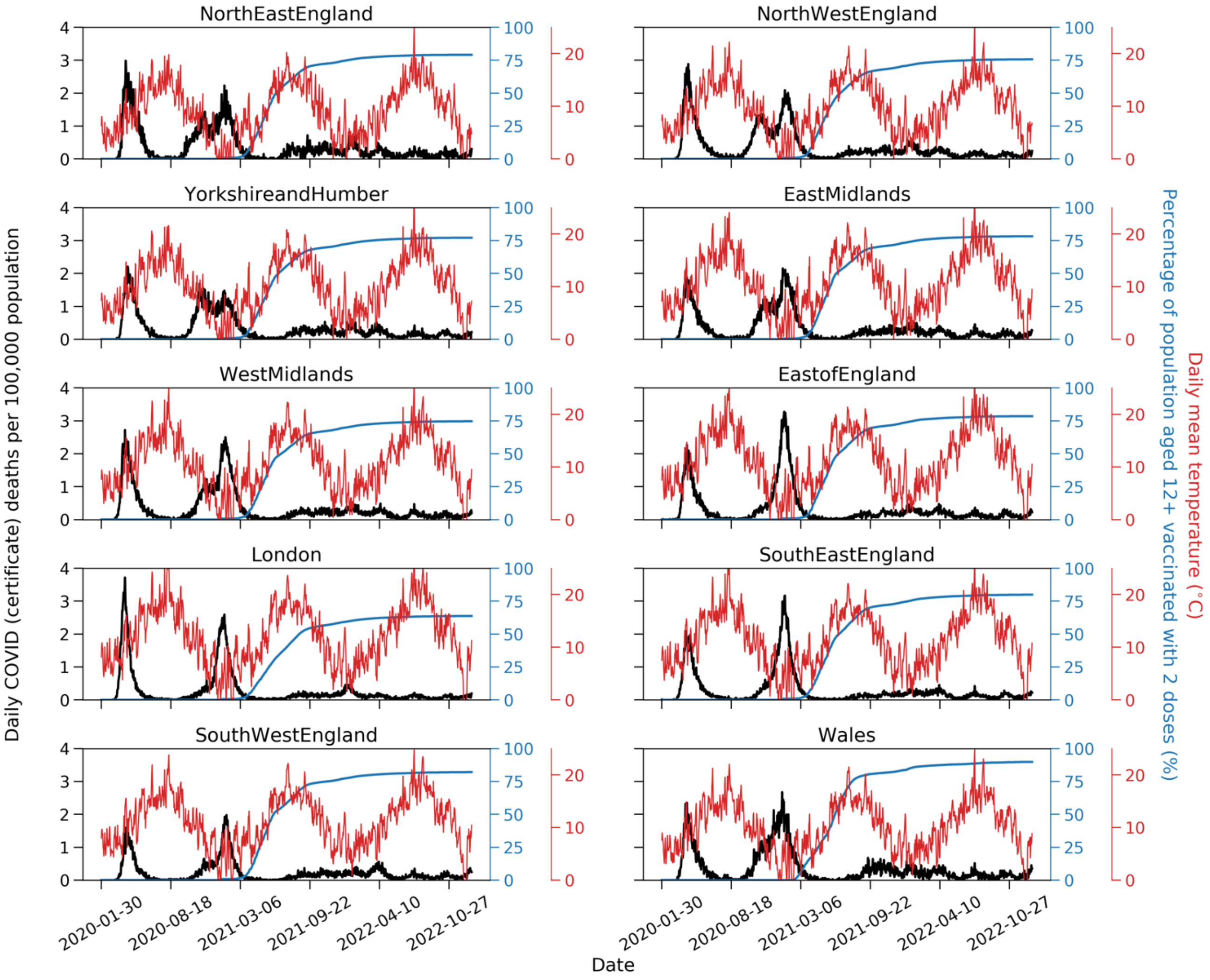

Figure S1. Time series of daily COVID-19 deaths, daily mean temperature, and cumulated COVID-19 vaccination for all study regions. The black lines show daily COVID-19 death occurrences indicated by death certificates, per 100,000 people in each region in England and Wales. The red lines show daily mean temperatures, and the blue lines show regional cumulated percentage of population aged 12+ who have had 2 doses of COVID-19 vaccines. This figure covers the study period, 30 January 2020 to 31 December 2022.

Table S1. The cumulative percentage of people aged 12 and above who have received two doses of COVID-19 vaccines as of 1 July 2021. The data are displayed by regions in England and Wales.

Region	Vaccine uptake (%)	Region	Vaccine uptake (%)
North East England	53.9	North West England	50.9
Yorkshire and Humber	51.8	East Midlands	53.1
West Midlands	50	East of England	52.1
London	35.9	South East England	51.8
South West England	55	Wales	61.6

Table S2. Start and end dates of heatwaves and cold snaps between 30 January 2020 and 31 December 2022. All dates are in dd/mm/yyyy. "--" indicates no additional events that year.

	Heatwaves		Cold snaps	
Year	Start date	End date	Start date	End date
2020	23/06/2020	27/06/2020	29/12/2020	18/01/2021
	30/07/2020	01/08/2020	--	--
	05/08/2020	15/08/2020	--	--
2021	16/07/2021	23/07/2021	22/01/2021	02/02/2021
	06/09/2021	09/09/2021	08/02/2021	12/02/2021
	--	--	26/11/2021	29/11/2021
	--	--	20/12/2021	23/12/2021
2022	16/06/2022	19/06/2022	04/01/2022	10/01/2022
	10/07/2022	25/07/2022	13/01/2022	17/01/2022
	30/07/2022	05/08/2022	07/12/2022	18/12/2022
	08/08/2022	17/08/2022	--	--
	23/08/2022	25/08/2022	--	--

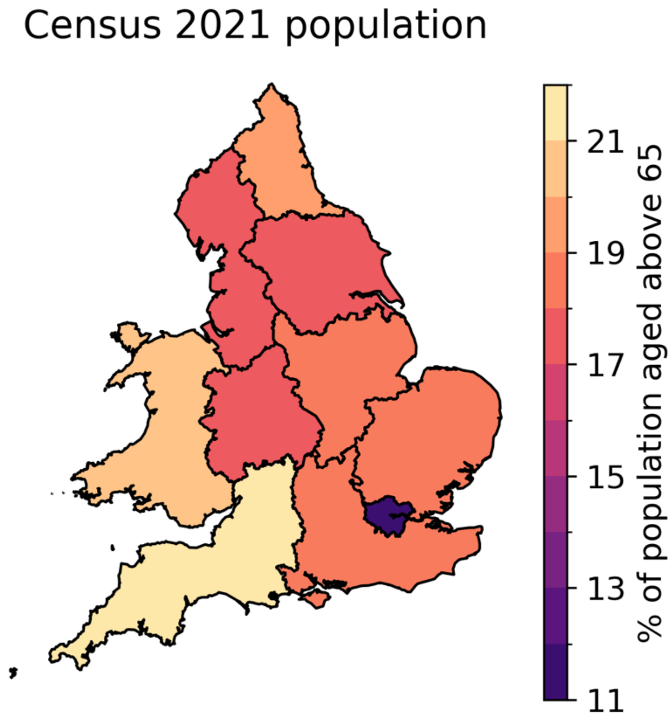

Figure S2. Percentage of population who are above the age of 65 in all studied regions. This figure is based on the 2021 Census.

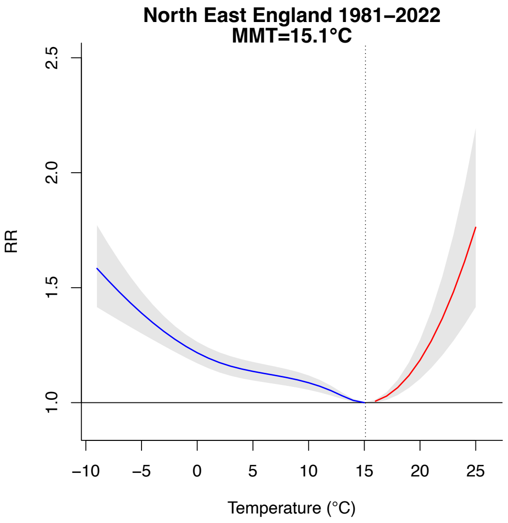
North East England 1981–2022
MMT=15.1°C
RR
Temperature (°C)

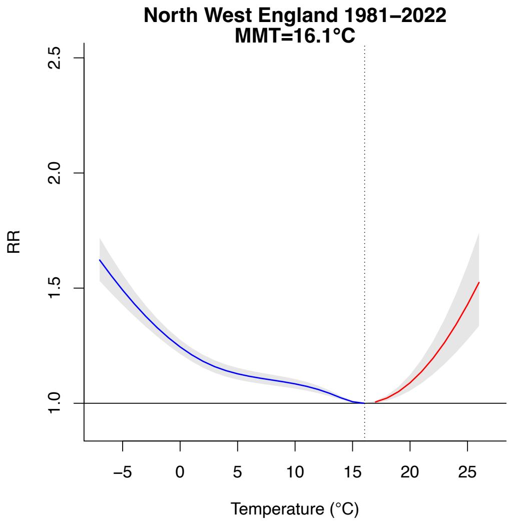
North West England 1981–2022
MMT=16.1°C
RR
Temperature (°C)

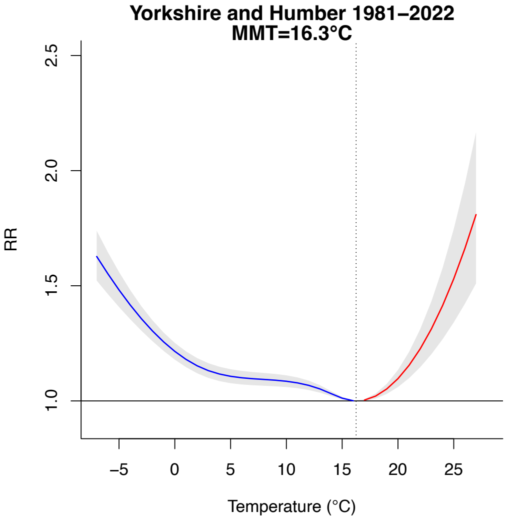
Yorkshire and Humber 1981–2022
MMT=16.3°C
RR
Temperature (°C)

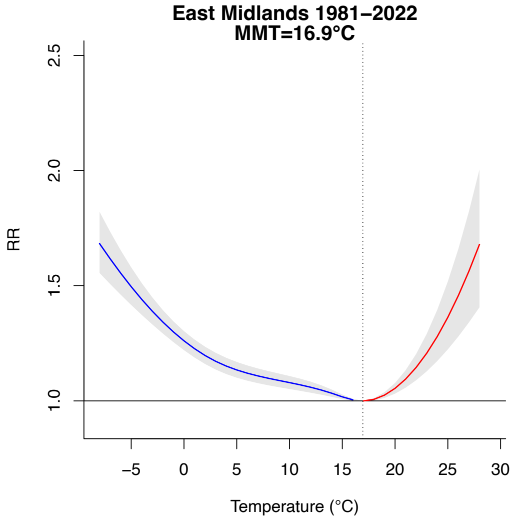
East Midlands 1981–2022
MMT=16.9°C
RR
Temperature (°C)

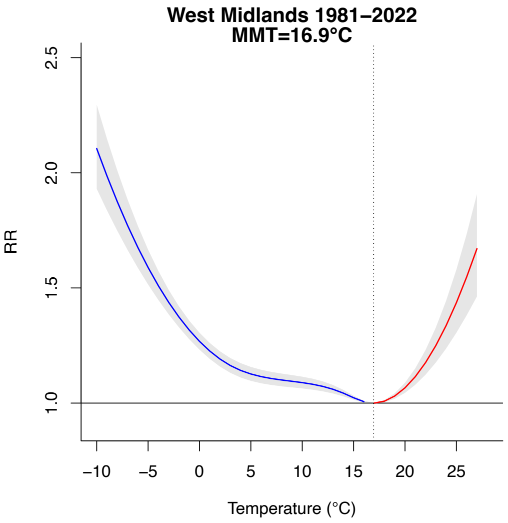
West Midlands 1981–2022
MMT=16.9°C
RR
Temperature (°C)

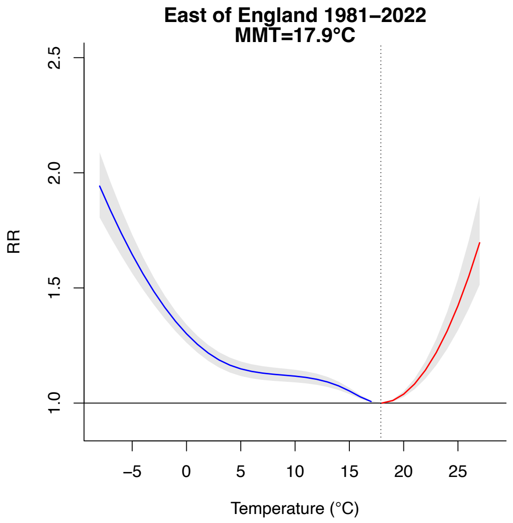
East of England 1981–2022
MMT=17.9°C
RR
Temperature (°C)

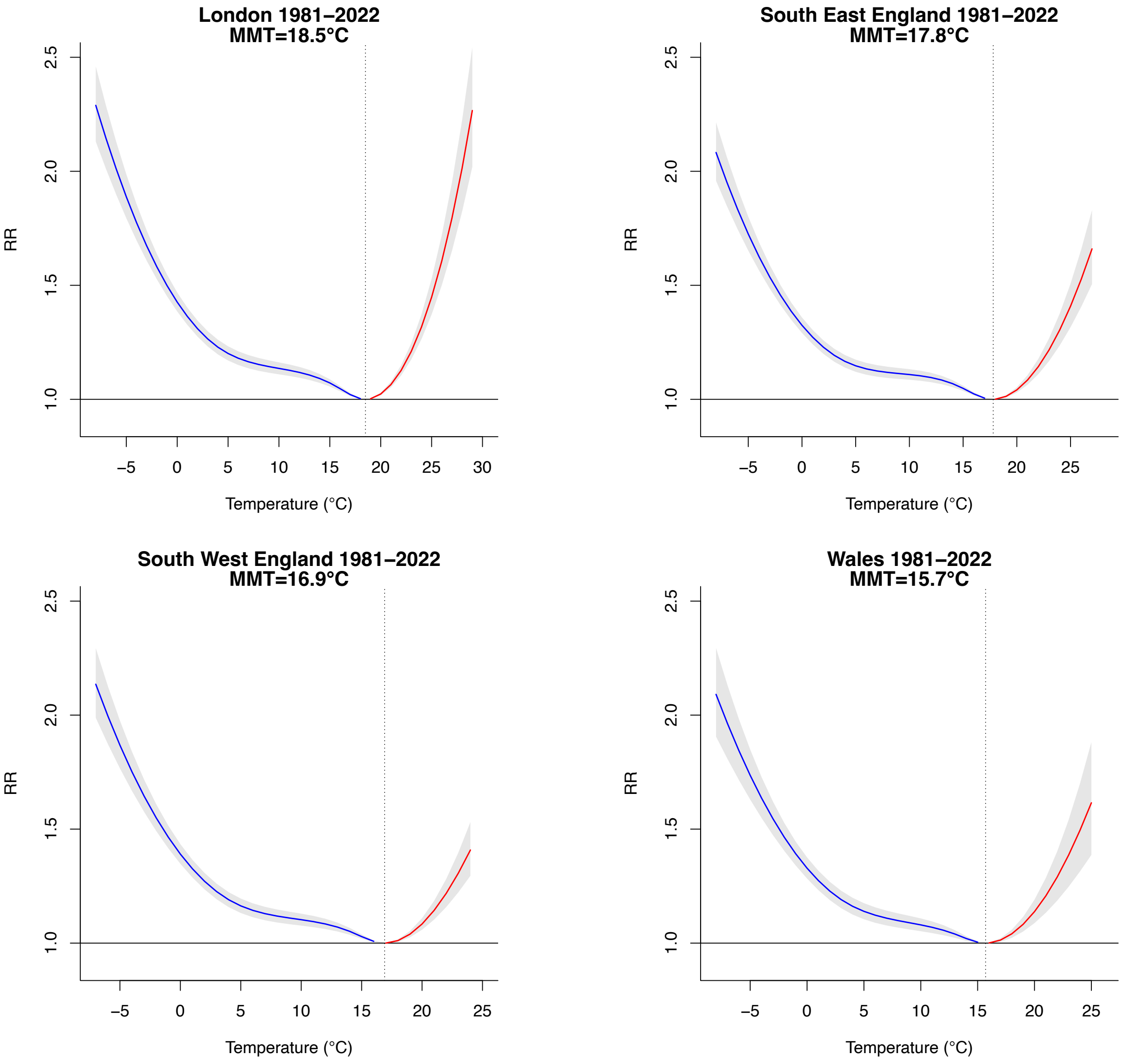

Figure S3. Temperature-mortality associations for all studied regions in the period 1981-2022. The y axis shows relative risk of mortality (RR), whereas the x axis indicates daily mean temperature. The grey shading shows the 95% confidence interval. The minimum mortality temperature (MMT) is shown at the top of each panel, as well as indicated by the grey vertical dashed line.

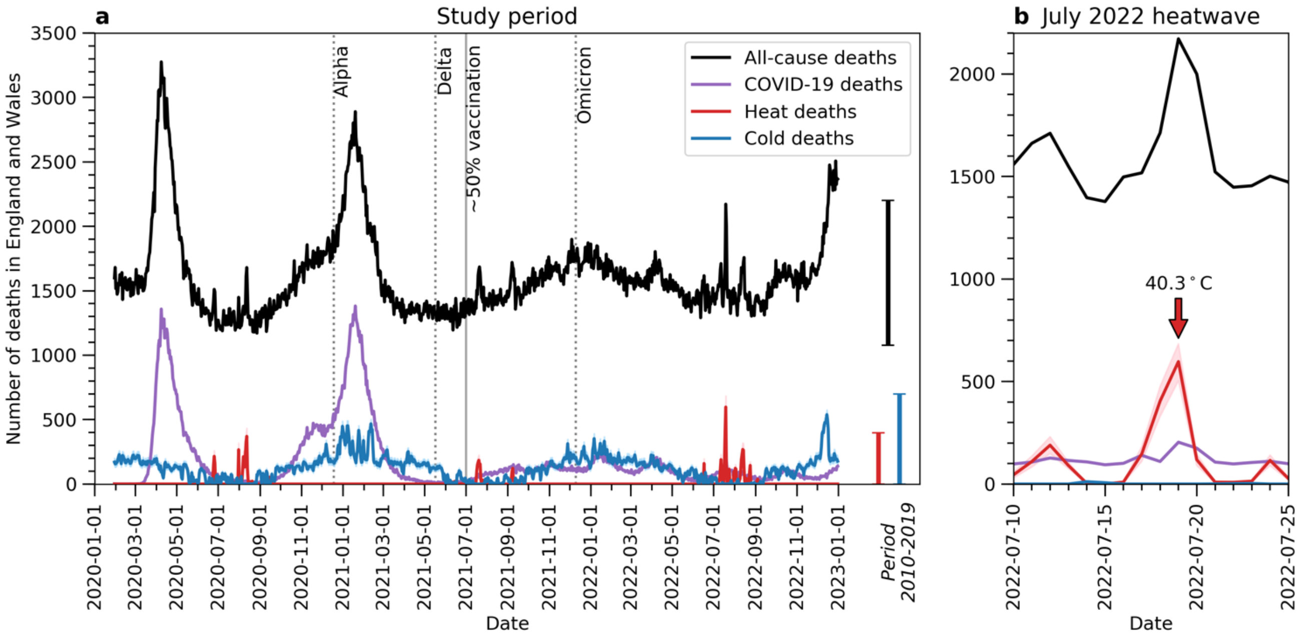

Figure S4. Same as Figure 1 in the main manuscript, but with heat and cold deaths estimated from population weighted average temperatures. Population weighted average temperatures were calculated from 25 km HadUK-Grid temperatures and 2021 Census Lower layer Super Output Area population aggregated to the 25 km grid.

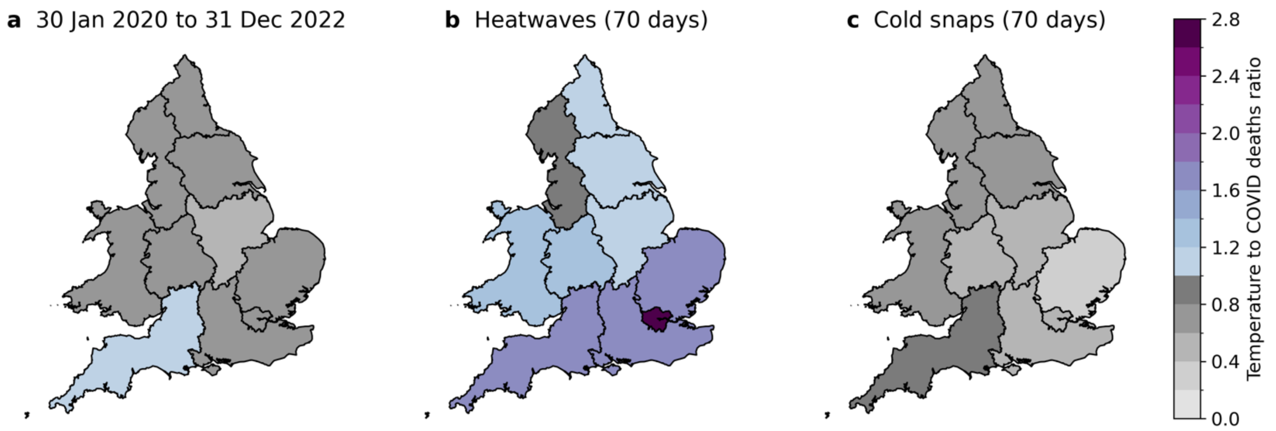

Figure S5. Same as Figure 2 in the main manuscript, but with temperature-related deaths estimated from population weighted regional average temperatures. Population weighted average temperatures were calculated from 25 km HadUK-Grid temperatures and 2021 Census Lower layer Super Output Area population aggregated to the 25 km grid.

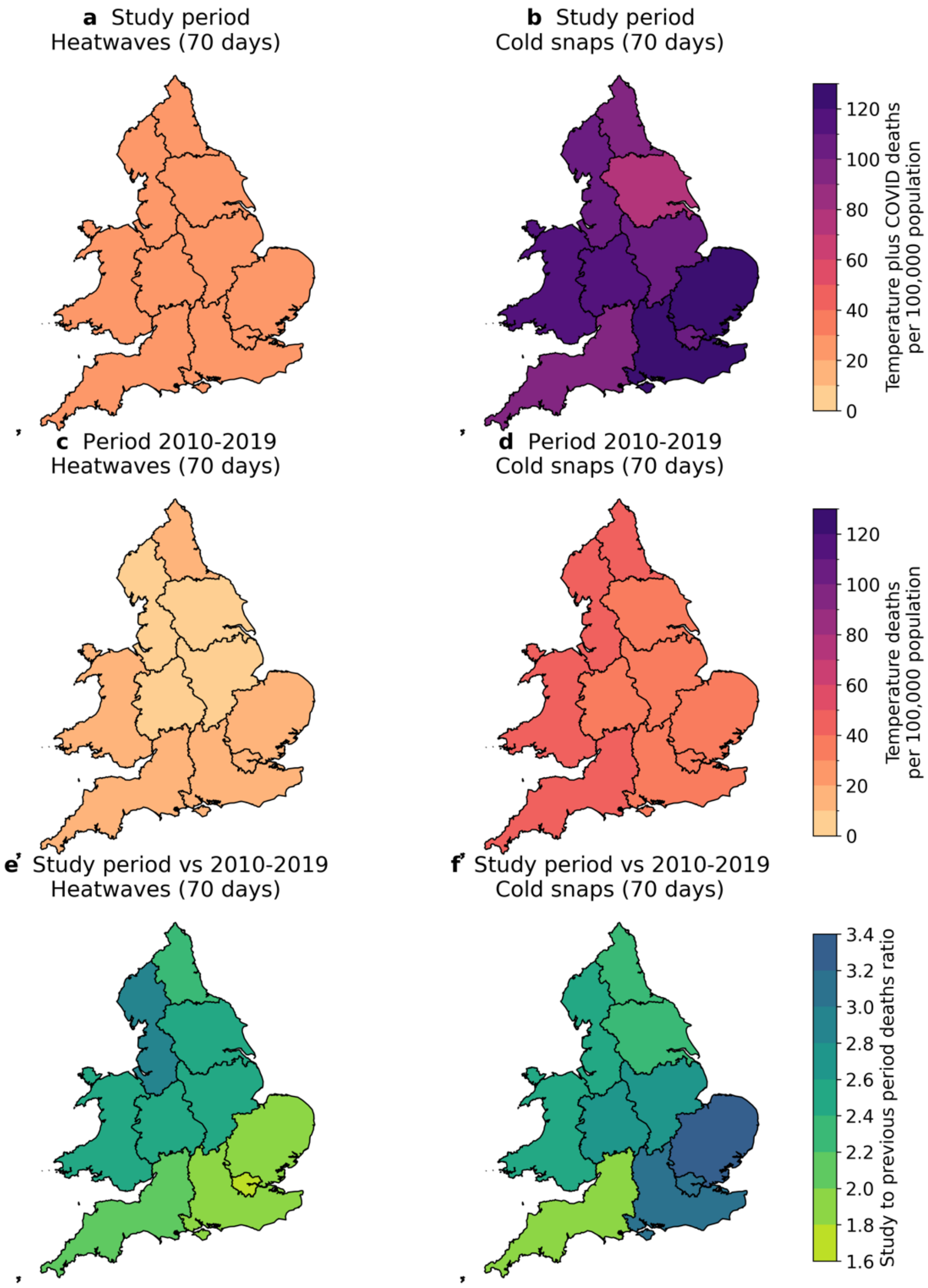

Figure S6. Same as Figure 3 in the main manuscript, but with temperature-related deaths estimated from population weighted regional average temperatures. Population weighted average temperatures were calculated from 25 km HadUK-Grid temperatures and 2021 Census Lower layer Super Output Area population aggregated to the 25 km grid.

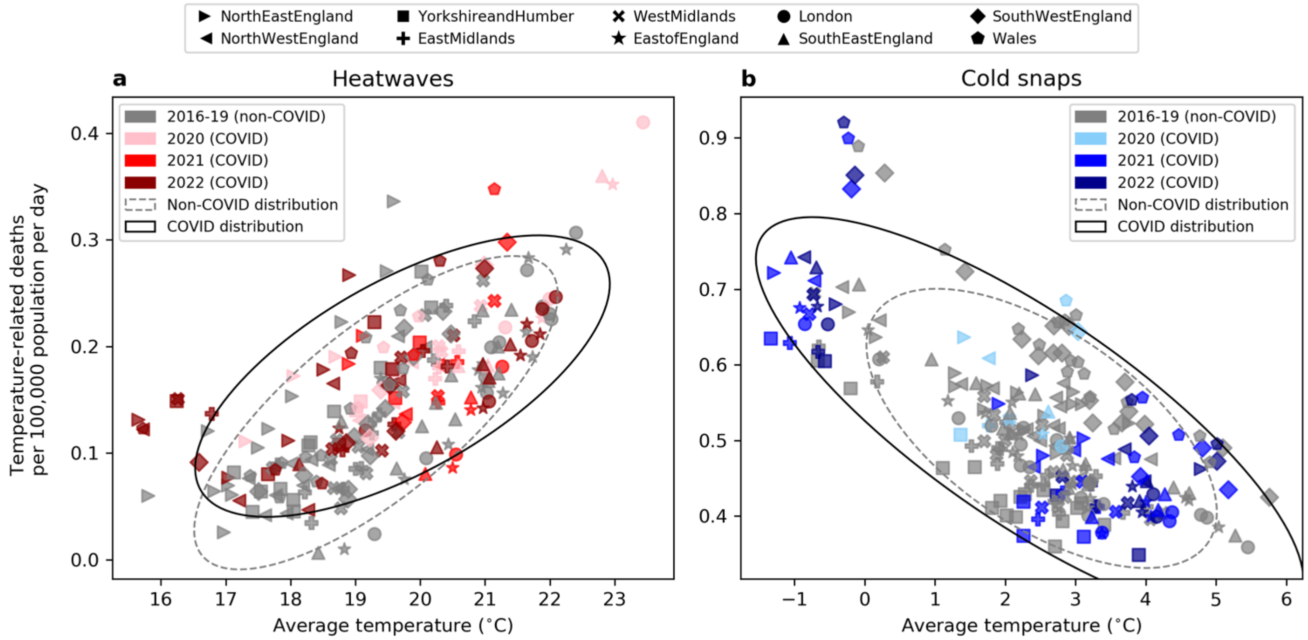

Figure S7. Same as Figure 4 in the main manuscript, but with temperature-related deaths estimated from population weighted regional average temperatures. Population weighted average temperatures were calculated from 25 km HadUK-Grid temperatures and 2021 Census Lower layer Super Output Area population aggregated to the 25 km grid.
