# Supplementary material for: Compound mortality impacts from extreme temperatures and the COVID-19 pandemic
Source: Nat Commun. 2024 May 23;15:4289. doi: 10.1038/s41467-024-48207-2 (PMC11116452; doi:10.1038/s41467-024-48207-2)
Supplement: Supplementary file 3 — Reporting Summary [file 41467_2024_48207_MOESM3_ESM.pdf]

nature portfolio

Corresponding author(s): Y T Eunice Lo

Last updated by author(s): Apr 18, 2024

Reporting Summary

Nature Portfolio wishes to improve the reproducibility of the work that we publish. This form provides structure for consistency and transparency in reporting. For further information on Nature Portfolio policies, see our Editorial Policies and the Editorial Policy Checklist.

Statistics

For all statistical analyses, confirm that the following items are present in the figure legend, table legend, main text, or Methods section.

n/a	Confirmed	
☒	☐	The exact sample size (*n*) for each experimental group/condition, given as a discrete number and unit of measurement
☒	☐	A statement on whether measurements were taken from distinct samples or whether the same sample was measured repeatedly
☐	☒	The statistical test(s) used AND whether they are one- or two-sided<br>*Only common tests should be described solely by name; describe more complex techniques in the Methods section.*
☒	☐	A description of all covariates tested
☒	☐	A description of any assumptions or corrections, such as tests of normality and adjustment for multiple comparisons
☐	☒	A full description of the statistical parameters including central tendency (e.g. means) or other basic estimates (e.g. regression coefficient) AND variation (e.g. standard deviation) or associated estimates of uncertainty (e.g. confidence intervals)
☐	☒	For null hypothesis testing, the test statistic (e.g. *F*, *t*, *r*) with confidence intervals, effect sizes, degrees of freedom and *P* value noted<br>*Give P values as exact values whenever suitable.*
☒	☐	For Bayesian analysis, information on the choice of priors and Markov chain Monte Carlo settings
☒	☐	For hierarchical and complex designs, identification of the appropriate level for tests and full reporting of outcomes
☒	☐	Estimates of effect sizes (e.g. Cohen's *d*, Pearson's *r*), indicating how they were calculated

Our web collection on statistics for biologists contains articles on many of the points above.

Software and code

Policy information about availability of computer code

Data collection	No software was used.
Data analysis	Python 3.6.3 to 3.12.1 and R 3.5.2. The code is available at https://github.com/BrisClimate/Compound-temps-covid-mortality-paper.

For manuscripts utilizing custom algorithms or software that are central to the research but not yet described in published literature, software must be made available to editors and reviewers. We strongly encourage code deposition in a community repository (e.g. GitHub). See the Nature Portfolio guidelines for submitting code & software for further information.

Data

Policy information about availability of data

All manuscripts must include a data availability statement. This statement should provide the following information, where applicable:

- Accession codes, unique identifiers, or web links for publicly available datasets
- A description of any restrictions on data availability
- For clinical datasets or third party data, please ensure that the statement adheres to our policy

Met Office HadUK-Grid climate observations by administrative regions over the UK are available on the CEDA archive (https://catalogue.ceda.ac.uk/uuid/b39898e76ab7434a9a20a6dc4ab721f0). Daily all-cause death occurrences in regions of England and Wales are available from the Office for National Statistics (https://www.ons.gov.uk/peoplepopulationandcommunity/birthsdeathsandmarriages/deaths/adhocs/14173dailydeathsoccurrencesenglandandwales1981and2020 and https://www.ons.gov.uk/peoplepopulationandcommunity/birthsdeathsandmarriages/deaths/

adhocs/1724dailydeathoccurrencesenglandandwales2021and2022). Mid-2021 and mid-2015 population estimates for the UK are available from the Office for National Statistics (https://www.ons.gov.uk/peoplepopulationandcommunity/populationandmigration/populationestimates/datasets/populationestimatesforukenglandandwalesscotlandandnorthernireland). Daily deaths with COVID-19 on the death certificate by UK regions and nations can be obtained from the Government Coronavirus (COVID-19) in the UK dashboard (https://coronavirus.data.gov.uk/details/deaths). Cumulative COVID-19 vaccine uptake by dosage and regions were available on the dashboard (https://coronavirus.data.gov.uk/details/vaccinations), but it has since been discontinued. NHS England hospital bed availability and occupancy are available from NHS England Statistics (https://www.england.nhs.uk/statistics/statistical-work-areas/bed-availability-and-occupancy/). NHS Wales hospital bed availability and occupancy are available from StatsWales (https://statswales.gov.wales/Catalogue/Health-and-Social-Care/NHS-Hospital-Activity/NHS-Beds).

Research involving human participants, their data, or biological material

Policy information about studies with human participants or human data. See also policy information about sex, gender (identity/presentation), and sexual orientation and race, ethnicity and racism.

Reporting on sex and gender	Not applicable as the analysis is not sex or gender stratified.
Reporting on race, ethnicity, or other socially relevant groupings	Not applicable as the analysis is not race, ethnicity, or other socially relevant groupings stratified.
Population characteristics	The percentage of population aged above 65 is reported in Supplementary Information for all studied regions. Diagnosis or other characteristics are not applicable.
Recruitment	There was no recruitment.
Ethics oversight	All health data used were publicly available.

Note that full information on the approval of the study protocol must also be provided in the manuscript.

Field-specific reporting

Please select the one below that is the best fit for your research. If you are not sure, read the appropriate sections before making your selection.

☐ Life sciences ☐ Behavioural & social sciences ☒ Ecological, evolutionary & environmental sciences

For a reference copy of the document with all sections, see nature.com/documents/nr-reporting-summary-flat.pdf

Ecological, evolutionary & environmental sciences study design

All studies must disclose on these points even when the disclosure is negative.

Study description	Quantitative data are described in the manuscript.
Research sample	All sexes and ages in England and Wales are included. The data and source of all pre-existing datasets are described in Methods.
Sampling strategy	No sample size calculation was performed because we wanted to study the whole population.
Data collection	Mortality data were obtained from the Office for National Statistics and Gov.UK. Temperature data were obtained from the Met Office.
Timing and spatial scale	The analysis spans 2010 to 2022. Frequency of the publicly available data is daily. These are detailed in the manuscript.
Data exclusions	No data were excluded.
Reproducibility	All analyses are reproducible with the publicly available data and the provided computer code.
Randomization	This is not relevant to this study because it focuses on population-level mortality.
Blinding	No individual's information were available in the pre-existing dataset.

Did the study involve field work? ☐ Yes ☒ No

Reporting for specific materials, systems and methods

We require information from authors about some types of materials, experimental systems and methods used in many studies. Here, indicate whether each material, system or method listed is relevant to your study. If you are not sure if a list item applies to your research, read the appropriate section before selecting a response.

Materials & experimental systems

n/a	Involved in the study	
☒	☐	Antibodies
☒	☐	Eukaryotic cell lines
☒	☐	Palaeontology and archaeology
☒	☐	Animals and other organisms
☒	☐	Clinical data
☒	☐	Dual use research of concern
☒	☐	Plants

Methods

n/a	Involved in the study	
☒	☐	ChIP-seq
☒	☐	Flow cytometry
☒	☐	MRI-based neuroimaging

Plants

Seed stocks	No seed stocks were involved.
Novel plant genotypes	No plants were involved.
Authentication	No plants or seed stock were involved.
